# Deep Sequencing of HPV16 E6 Region Reveals Unique Mutation Pattern of HPV16 and Predicts Cervical Cancer

**DOI:** 10.1128/spectrum.01401-22

**Published:** 2022-06-23

**Authors:** Wenchao Ai, Chuanyong Wu, Liqing Jia, Xiao Xiao, Xuewen Xu, Min Ren, Tian Xue, Xiaoyan Zhou, Ying Wang, Chunfang Gao

**Affiliations:** a Department of Laboratory Medicine, Shanghai Eastern Hepatobiliary Surgery Hospital, Shanghai, China; b Clinical Laboratory Medicine Center, Yueyang Hospital of Integrated Traditional Chinese and Western Medicine, Shanghai University of Traditional Chinese Medicine, Shanghai, China; c Department of Pathology, Fudan University Shanghai Cancer Center, Fudan University, Shanghai, China; Johns Hopkins Hospital

**Keywords:** cervical cancer, human papillomavirus type 16, next-generation sequencing, machine learning, E6 oncoprotein

## Abstract

The genetic diversity of human papillomavirus (HPV) 16 within cervical cells and tissue is usually associated with persistent virus infection and precancerous lesions. To explore the HPV16 mutation patterns contributing to the cervical cancer (CC) progression, a total of 199 DNA samples from HPV16-positive cervical specimens were collected and divided into high‐grade squamous intraepithelial lesion (HSIL) and the non‐HSIL(NHSIL) groups. The HPV16 E6 region (nt 7125-7566) was sequenced using next-generation sequencing. Based on HPV16 E6 amino acid mutation features selected by Lasso algorithm, four machine learning approaches were used to establish HSIL prediction models. The receiver operating characteristic was used to evaluate the model performance in both training and validation cohorts. Western blot was used to detect the degradation of p53 by the E6 variants. Based on the 13 significant mutation features, the logistic regression (LR) model demonstrated the best predictive performance in the training cohort (AUC = 0.944, 95% CI: 0.913–0.976), and also achieved a high discriminative ability in the independent validation cohort (AUC = 0.802, 95% CI: 0.601–1.000). Among these features, the E6 D32E and H85Y variants have higher ability to degrade p53 compared to the E6 wildtype (*P* < 0.05). In conclusion, our study provides evidence for the first time that HPV16 E6 sequences contain vital mutation features in predicting HSIL. Moreover, the D32E and H85Y variants of E6 exhibited a significantly higher ability to degrade p53, which may play a vital role in the development of CC.

**IMPORTANCE** The study provides evidence for the first time that HPV16 E6 sequences contain vital mutation features in predicting the high‐grade squamous intraepithelial lesion and can reduce even more unneeded colposcopies without a loss of sensitivity to detect cervical cancer. Moreover, the D32E and H85Y variants of E6 exhibited a significantly higher ability to degrade p53, which may play a vital role in the development of cervical cancer.

## INTRODUCTION

HPV-induced cancers, particularly cervical cancer (CC), are expected to remain a significant global health challenge for the following decades (1). CC is the sixth most common cancer in Chinese women, with the development of 109,741 new cases and 59,060 deaths reported annually (2). It has been widely shown that persistent high-risk human papillomavirus (HPV) infections are the main risk factor for developing CC and its precursor lesions (3). As the most deleterious type, HPV16 was the leading cause of more than half of CC cases worldwide and has been detected in 50–55% of invasive CC cases (4, 5).

The direct colposcopy referral to of all women with positive results for the HPV16/18 genotyping test has been broadly suggested as a triage strategy of HPV-based screening programs (6). However, the major limitation of HPV16/18 genotyping tests is that we are unable to differentiate between transient and persistent infections. More than 90% of HPV16/18 infections will be cleared automatically by their innate immunity within a few years (7, 8). Just a limited proportion of patients with persistent infection of high-risk genotypes are at risk of developing tumors. Therefore, in order to reduce the burden of colposcopy referrals and related complications, a classification strategy is critical for patients who do not need colposcopy (9).

Multiple studies have revealed that the HPV variant has significant differences in the risk of HPV persistent infection and progression to cervical intraepithelial neoplasia (CIN) and CC (10–12). Meanwhile, several studies have analyzed the effect of HPV16 E6 oncoprotein variants overexpression in primary cultures of keratinocytes and found differences in their ability to induce serum/calcium resistant colonies and downregulation of p53/Bax (13), affecting several important cellular processes, including differentiation, apoptosis (14, 15), immortalization, transformation (16, 17), migration, and metastasis (17). Several studies have focused on the characterization of HPV16 gene variants in clinical samples obtained by traditional sanger sequencing methods (18–21). However, compared with the high sensitivity and specificity of the next-generation sequencing (NGS) (22), this method cannot accurately describe the complex sequence patterns of virus. The identification between HPV16-related high‐grade squamous intraepithelial lesion (HSIL) and non‐HSIL (NHSIL) based on the HPV16 E6 region using big data of NGS technology is still unexplored.

Previously, we have proposed a method to predict hepatocellular carcinoma using machine learning models based on HBV rt/s gene pattern features derived from NGS data (23, 24). In this study, we aimed to detect the E6 region of HPV16 positive patients by NGS to obtain the amino acid (aa) mutation features for individual HSIL prediction. Besides, to assess the carcinogenicity of the virus, we evaluate the differences in the degradation of p53 by HPV16 E6 mutations obtained from NGS.

## RESULTS

### Different HPV16 E6 feature characteristics between HSIL and NHSIL patients.

**FIG 1:**
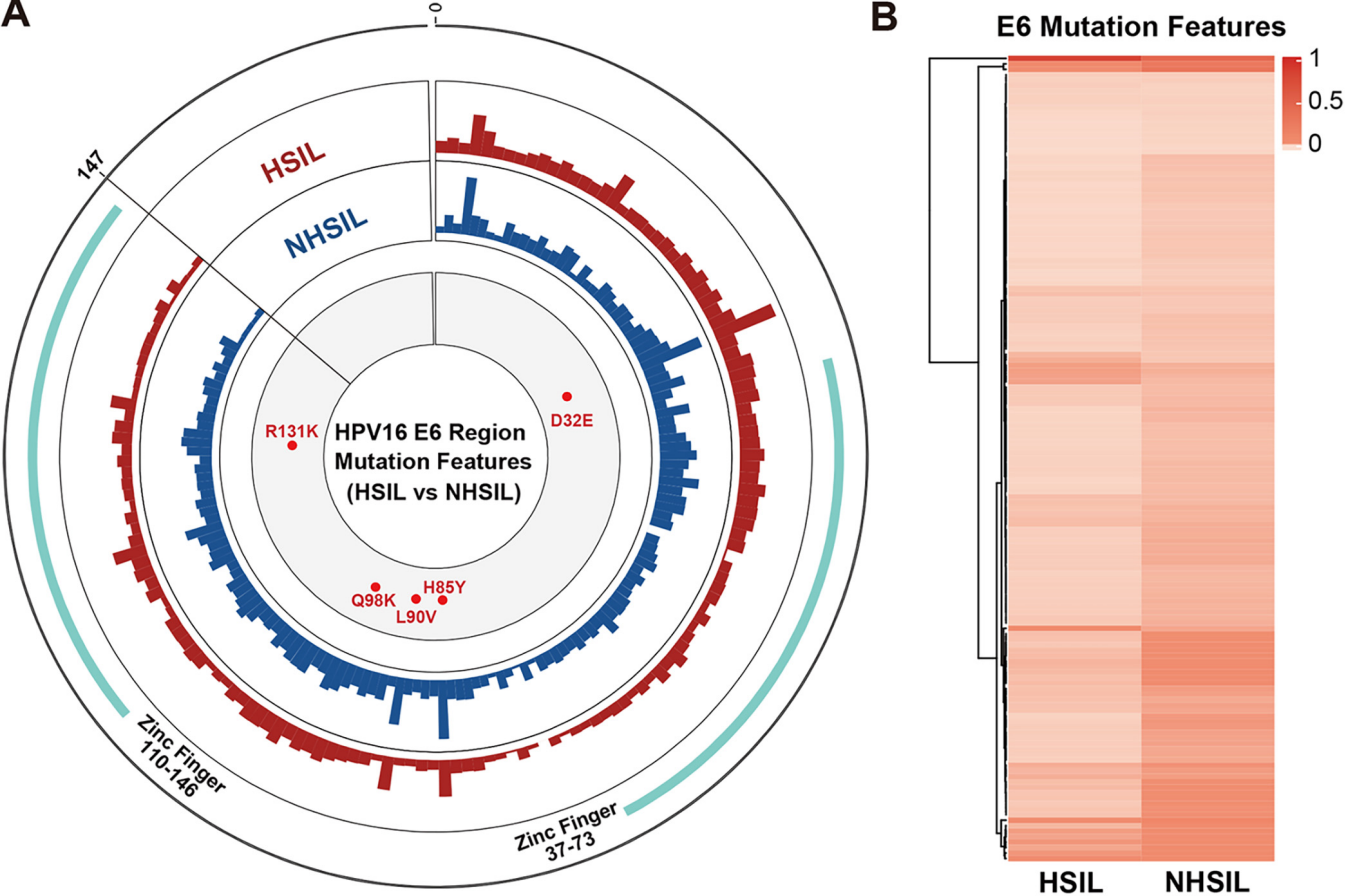
The complexity of the HPV16 E6 region between HSIL and NHSIL patients. (A) The outermost circle represents the location of amino acids (aa) of the HPV16 E6 protein (aa1–147). The important functional HPV16 E6 region distribution is marked as light green and as Zinc Finger (aa37–73 and aa110–146) (41). The colored histograms in the second and third circles indicate the complexity of mutation features (red, HSIL; blue, NHSIL) for each amino acid. The histogram represents the mutation features value. The innermost circle shows the differential aa mutations (red) between HSIL and NHSIL patients. (B) Different HPV16 E6 region mutation features among HSIL and NHSIL patients by hierarchical clustering. The value in the clustering map represents the mutation features of each group. A correlation was used for the sample measurement, Manhattan distance for the feature measurement, and ward.D2 algorithms for the clustering method.

HPV16 E6 mutation features were compared between HSIL and NHSIL patients based on the amino acid (aa) sequence of the HPV16 E6 gene (aa1–147) (Fig. 1A). The result showed that mutation features of the NHSIL patients were higher than those of HSIL patients in the E6 fragment, especially in the zinc finger (aa37–73 and aa110–146) regions (Fig. 1A). The cluster heatmap results were consistent with the circus plot. The E6 mutation features were different between HSIL and NHSIL patients, and the mutation frequency of HSIL patients was lower than that of NHSIL patients in general (Fig. 1B). We found that only 12 amino acid sites (1M, 5R, 8M, 12P, 17R, 27Q, 32D, 36E, 37C, 100N, 133H, and 144M) have a higher average mutation frequency in HSIL patients (Table S2 in the supplemental material). Meanwhile, five significantly different mutation sites (D32E, H85Y, L90V, Q98K, and R131K) between HSIL and NHSIL were identified using the unpaired Wilcoxon test and Fisher's exact test (Fig. 1 and Table S3). The above results indicated that great discrepancies were observed in mutation feature patterns between the HSIL and NHSIL patients.

### Screening significant mutation features for model construction.

**FIG 2:**
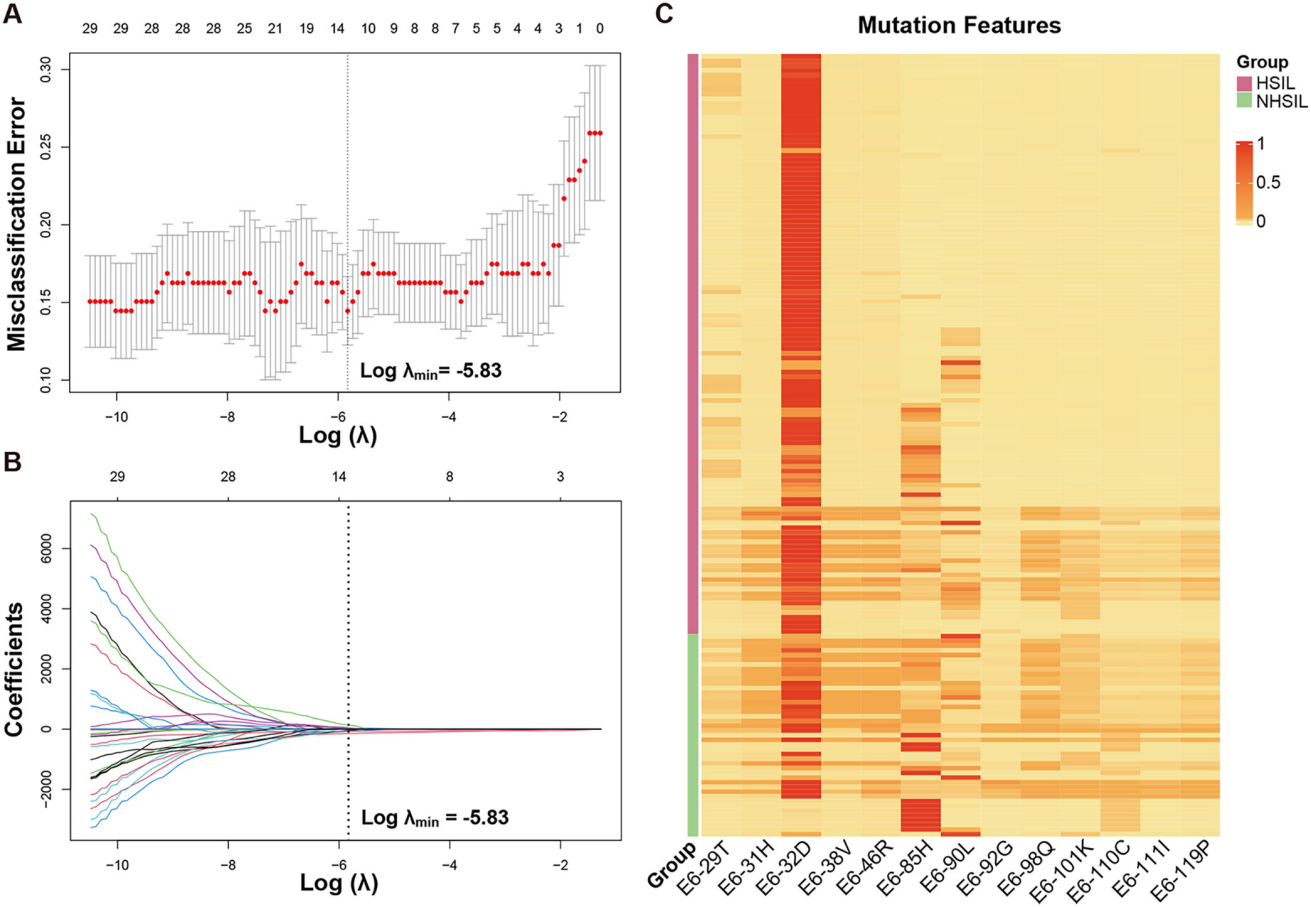
The dimensionality reduction results of HPV16 E6 mutation features by LASSO. (A, B) The coefficients in the Lasso regression for E6 mutation features screening. (A) For determining the adjustment parameter (λ value), we selected the adjustment parameter (λ value) with the minimum value of the misclassification error using the cross-validation method. (B) The adjustment parameter (λ value) was used to obtain the fraction deviance explained value. The obtained fraction deviance explained value was used to determine the final model selection. (C) Different mutation features among HSIL and NHSIL patients by hierarchical clustering.

When the missense mutation features of HPV16 E6 in HSIL and NHSIL patients were compared, a total of 33 differential mutation features were found (both Wilcoxon and Fisher’s exact tests *P* < 0.05). Subsequently, we used the Lasso regression algorithm to select the most significant mutation features for classifying HSIL and NHSIL at the minimum value of the misclassification error using the cross-validation method (Fig. 2A and B). In total, 13 mutation features were filtered. The mutation features obtained by Lasso exhibited very different patterns between HSIL and NHSIL (Fig. 2C).

### HSIL status prediction using machine learning approaches.

**FIG 3:**
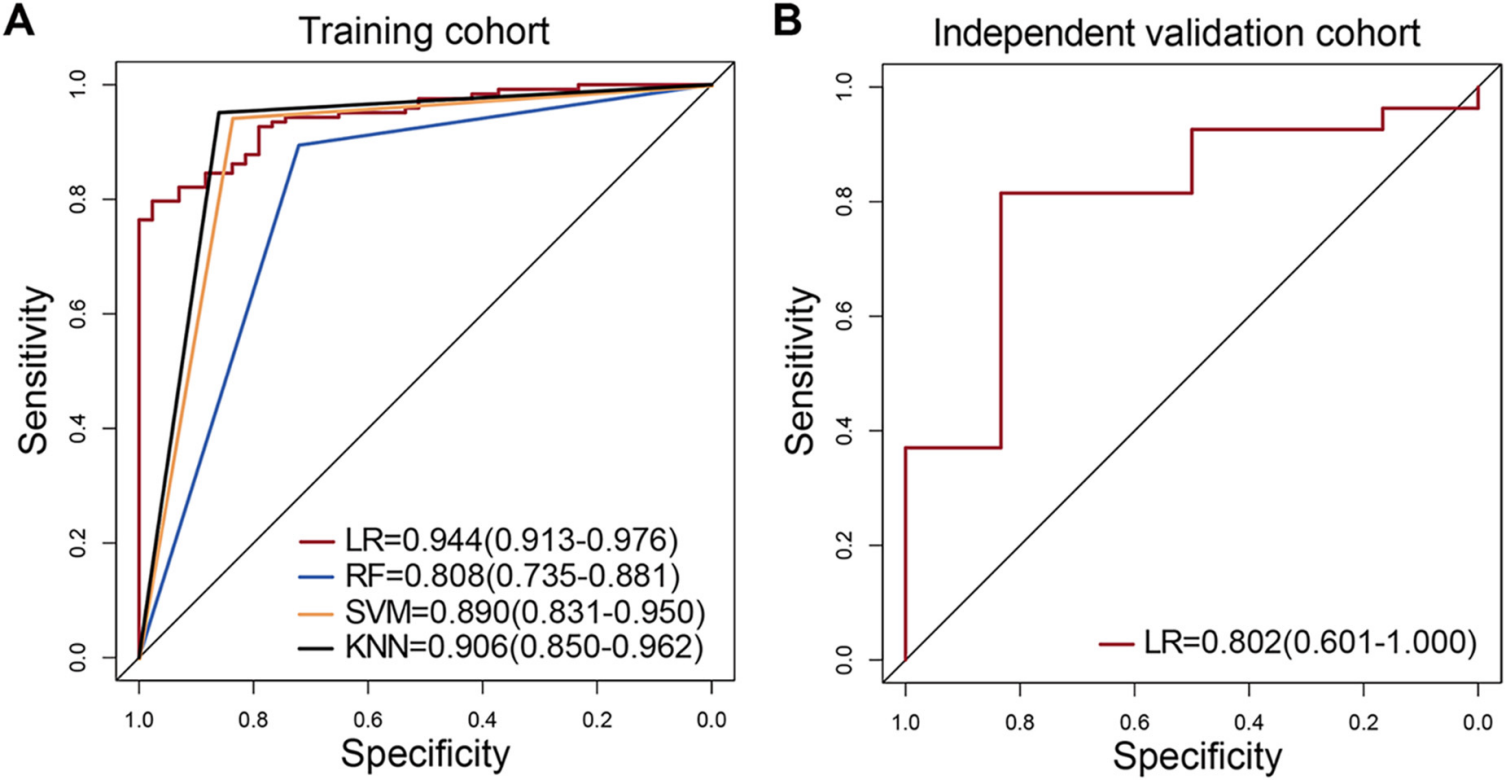
Four model performance in the training and validation cohorts. (A) Comparison of the AUC values between 4 models based on risk scores (red, LR; blue, RF; yellow, SVM; dark, KNN) in the training cohorts. (B) The AUC values of the LR model in the independent validation cohorts. The 95% confidence interval of AUC is also shown in the legend area.

Based on these 13 significant features by the Lasso algorithm, we then constructed the prediction models with the four machine learning approaches: logistic regression (LR), random forest (RF), support vector machine (SVM), and K-nearest neighbor (KNN). The area under the ROC curves (AUC) were used to evaluate their performance through 5-fold cross-validation in the training cohort (HSIL: *n* = 123 and NHSIL: *n* = 43). As shown in Fig. 3A, we found that the mutation features could accurately classify HSIL and NHSIL. It was worth noting that the LR model had a discriminating power of AUC values based on the mutation features in the training cohort (AUC = 0.944, 95% CI: 0.913–0.976) (Fig. 3A). For the independent validation cohort (HSIL: *n* = 27 and NHSIL: *n* = 6), the LR model also showed a satisfactory prediction performance (AUC = 0.802, 95% CI: 0.601–1.000) (Fig. 3B). The specificity and sensitivity of the LR model in differentiating HSIL from NHSIL were 83.3% and 81.9% in the independent validation cohort, respectively (Table 1). Thus, the LR algorithm based on the E6 mutation features achieved the best prediction performance in both training and validation cohorts.

**TABLE 1:** Prediction performance of the machine learning models in the training and validation cohorts^*a*^

Cohort	Model	AUC (95% CI)	Accuracy (%)	Specificity (%)	Sensitivity (%)	PPV (%)	NPV (%)
Training cohort	LR	0.944 (0.913–0.976)	84.3	97.7	79.7	99.0	62.7
	RF	0.808 (0.735–0.881)	84.9	72.1	89.4	90.1	70.5
	SVM	0.890 (0.831–0.950)	91.6	83.7	94.1	94.3	83.7
	KNN	0.906 (0.850–0.962)	92.8	86.1	95.1	95.1	86.1
							
Independent validation cohort	LR	0.802 (0.601–1.000)	81.8	83.3	81.9	95.7	50.0

a LR, logistic regression; RF, random forest; SVM, support vector machine; KNN, K-nearest neighbor; AUC, area under curves; CI, confidence interval; PPV, positive predictive value; NPV, negative predictive value.

### The 3D structural analysis of HPV16 E6 mutations.

**FIG 4:**
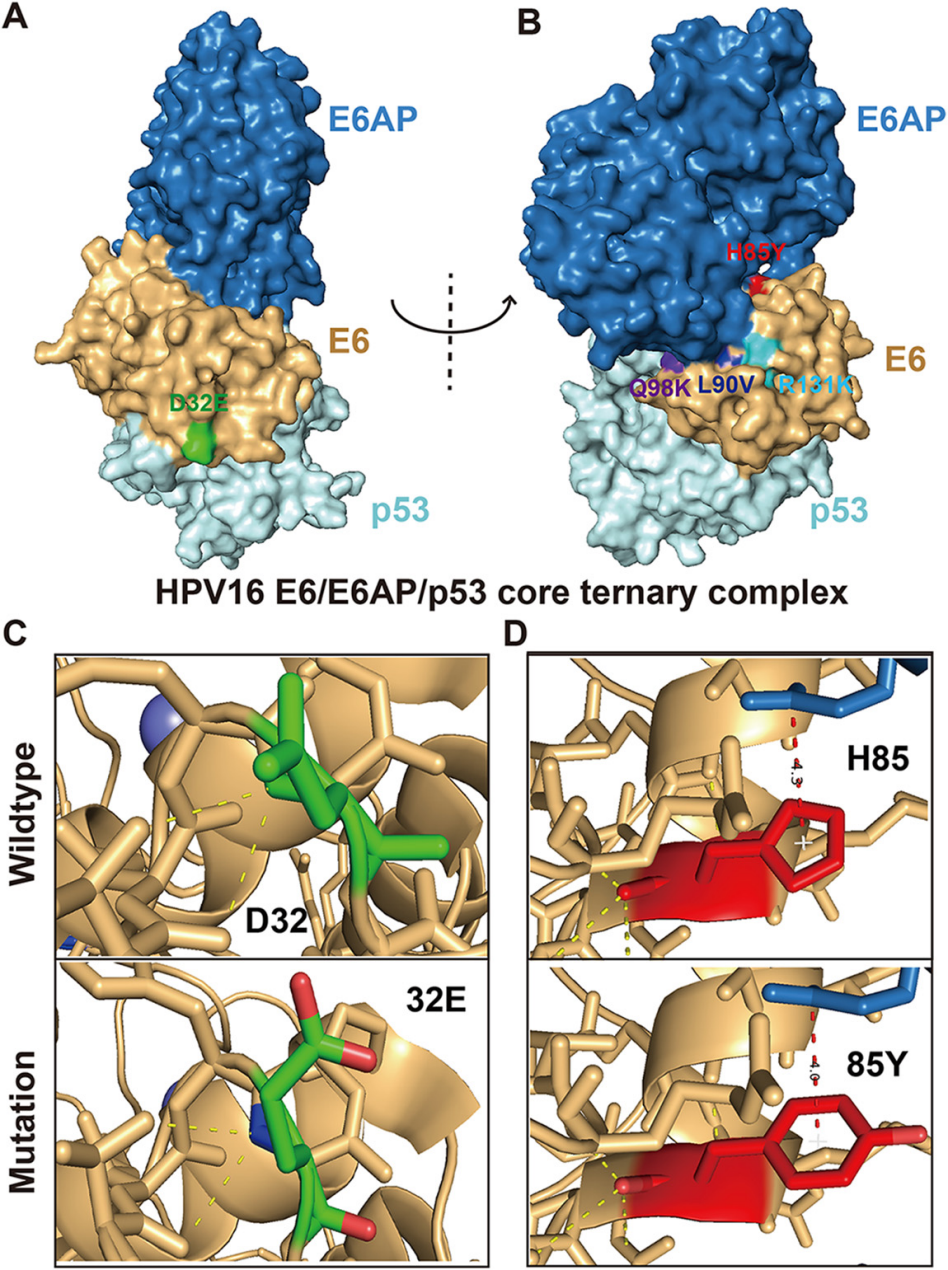
The 3D structural analysis of HPV16 E6 mutations. (A, B) Structure of the HPV16 E6/E6AP/p53 core ternary complex (25) (blue, E6AP peptide; brown, HPV16 E6; light cyan, p53 core). The mutations are marked on the surface by different colors (green, D32E; red, H85Y; dark blue, L90V; purple, Q98K; powder blue, R131K). (C) The 3D prediction structures of E6–p53 interface associated with D32E mutation. (D) The 3D prediction structures of E6–E6AP interface associated with H85Y mutation.

Further analysis on the 13 mutation features identified by Lasso algorithm revealed the significant differences in D32E, H85Y, L90V, and Q98K mutations between HSIL and NHSIL patients. Previous studies have shown that HPV16 oncoprotein E6 binds to a short Leucine-rich LxxLL consensus sequence within ubiquitin ligase E6AP, resulting in the E6/E6AP heterodimer leading to p53 degradation (25). In this study, the D32E mutations were adjacent to the E6‐p53 interface in the HPV16 E6/E6AP/p53 core ternary complex (Fig. 4A), while the H85Y, L90V, Q98K, and R131K mutations were found to be located near the E6‐E6AP interface (Fig. 4B), which may affect the E6‐p53 interaction.

### HPV16 E6 D32E and H85Y mutations drive enhanced degradation of p53.

**FIG 5:**
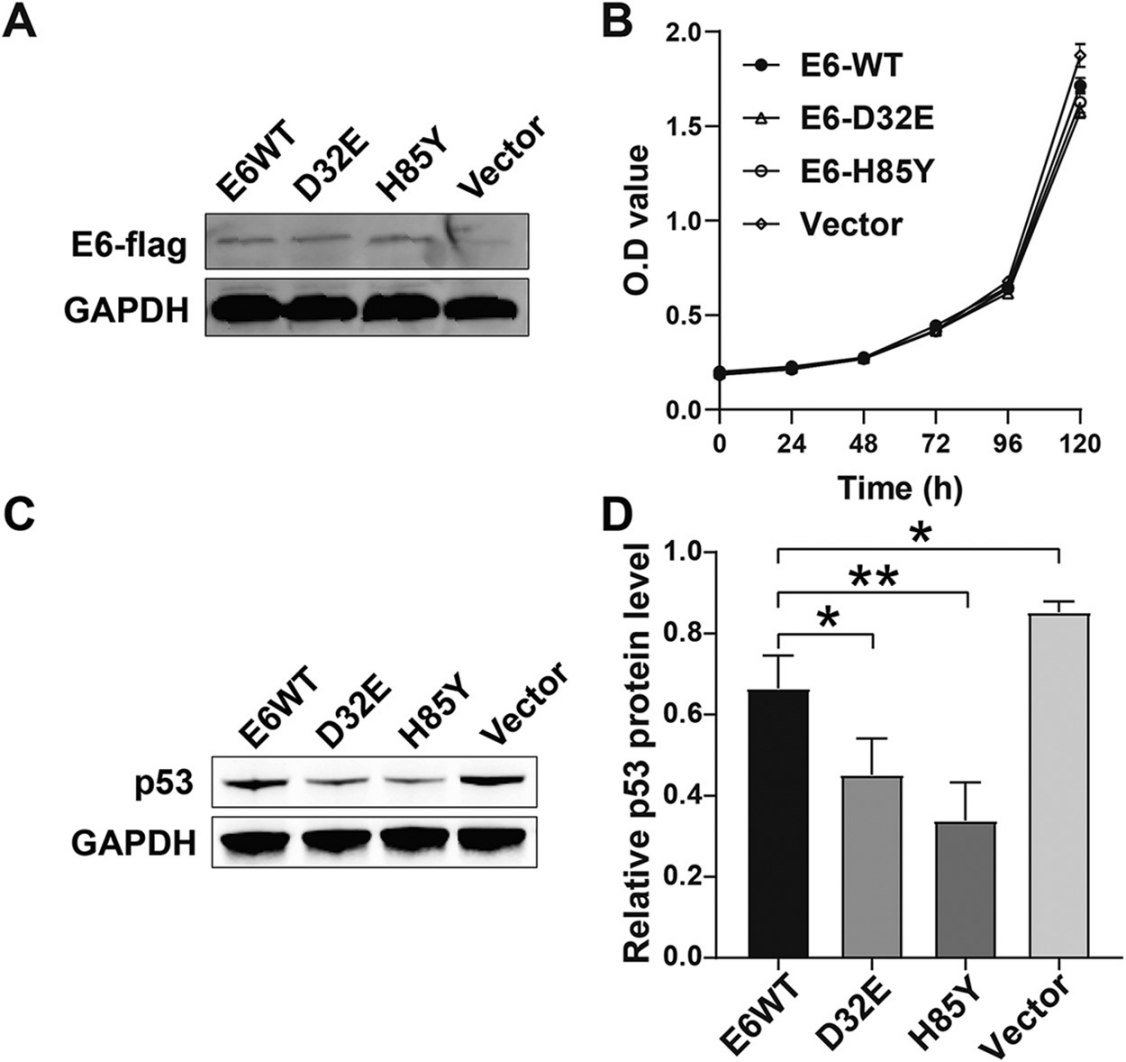
E6 variants (D32E and H85Y) drive enhanced degradation of p53. (A) E6-flag and GAPDH protein levels were assessed by Western blotting analysis. (B) CCK8 assay for exogenous HPV16 E6 expressing C-33A cells. (C, D) p53 and GAPDH protein levels were assessed by Western blotting analysis. GAPDH antibody was used as a loading control. *, *P* < 0.05; **, *P* < 0.01.

To analyze the effects of HPV16 E6 variants on cell phenotype and p53 degradation, we constructed plasmids of the E6 wild type (NP_041325.1) and E6 variants (D32E and H85Y) and expressed them in HPV-negative cervical cancer cell line. The cell proliferation assay showed that the D32E and H85Y variants had no effect on the cell proliferation (Fig. 5B). However, the Western blot results showed that both E6 wild type and E6 variants (D32E and H85Y) could degrade the p53 protein (Fig. 5C and D). And the D32E and H85Y variants exhibited a significantly higher ability to degrade p53 compared to the E6 wildtype (*P* < 0.05). Moreover, the H85Y variant was slightly more efficient in degrading p53 than the D32E variant (*P* < 0.01).

## DISCUSSION

High-risk HPV persistent infection is well known to be a major risk of cervical cancer, with 70% of persistent infections attributed to HPV16 and 18 (26). As the major oncoprotein of HPV, E6 is related to the cellular immortalization, malignant transformation, and carcinogenesis through its abilities to degrade p53 (17, 25). To explore the vital features in HPV16 DNA sequences that can be used for CC risk prediction and act as a further triage for HPV-based genotyping tests, we propose a machine learning-based algorithm to distinguish HSIL patients from NHSIL patients based on HPV16 E6 sequence features derived from NGS.

In this study, we comprehensively discuss the mutation characteristics of the HPV16 E6 region in HSIL and NHSIL patients based on NGS. The result showed that the HSIL patients exhibited very distinct mutation patterns at the E6 amino acid level compared to the NHSIL patients. For women with positive results for HPV16 genotyping, the NGS-based HPV16 E6 sequence analysis demonstrated better discrimination of related progressive infections, which could reduce the colposcopy referral burden and associated complications.

The results showed that the mutation frequency of HSIL patients was generally lower than that of NHSIL patients (Fig. 1A), which is consistent with the findings of the previous study (27). The more complicated NHSIL mutations may be induced, at least in part, by the antiviral activity of human APOBEC3 (hA3) cytidine deaminases (28). It has been demonstrated that hA3A-mediated cytidine deaminase activity is capable of inducing HPV16 mutations (29) and suppressing HPV infectivity (30). Warren et al. (30) also proposed that these induced mutations, if not fatal, may be the cause of the long-term accumulation of genomic changes that lead to HPV-associated cancers.

In addition, to verify whether the subgroups of HSIL and NHSIL patients have an impact on the differences in E6 mutation features, we investigated the E6 mutation features among these subgroups (Fig. S3). Six higher frequencies of missense mutations including R62I, L90V, Q98K, E120D, Q130R, and R131K were identified in the subgroups of CC compared to that of CIN2/3 (Table S4, both Wilcoxon and Fisher’s exact tests *P* < 0.05). However, only the E120D missense mutations showed significant difference between the subgroups low-grade squamous intraepithelial lesion (LSIL) and nonneoplastic/no evidence of disease (NED) (Table S4, *P* < 0.05). Between the subgroups of HSIL patients, the mutation features of zinc finger (aa37–73 and aa110–146) regions were higher in CIN2/3 than those in CC patients. However, this difference was not shown in the subgroups of NHSIL patients (Fig. S3). Our results showed that the more complex NHSIL mutations were indeed closely related to the low frequency mutation in HSIL patients (Fig. S3). In a previous study, Mirabello et al. (1) also evaluated rare variants in controls stratified according to cytological findings and found that HPV16-infected women with normal (NILM) cytology had significantly more variation than those with cytomorphologic manifestations of an HPV infection (LSIL, *P* = 0.01), suggesting that variability might be related to decreased fitness.

The different E6 mutation feature patterns prompted us to explore the feasibility of using these different features of HPV16 E6 sequences to predict the risk of HSIL. Therefore, four different machine learning models were applied in this study, including LR, RF, SVM, and KNN. Evaluation of the AUC values showed that the LR algorithm achieved an approving diagnostic performance in both the training [AUC = 0.944 (0.913–0.976)] and the independent validation cohort [AUC = 0.802 (0.601–1.000)], indicating the potential clinical applications for HSIL status prediction (Table 1).

Recent studies have shown that novel molecular assays for recognizing proliferating cells and methylated target host genes could be used to triage HPV-positive cases (31). The Mexico cervical cancer screening study trial showed that in women with positive results for HPV16/18 genotyping, the p16/*K_i_*-67 dual-stained cytology test performs better than cytology and E6 oncoprotein in discriminating relevant progressive infections (6). For the detection of CIN2+ lesions, the p16/*K_i_*-67 dual-stained cytology was found to have higher sensitivity than liquid-based cytology (55.2% versus 23.9%) but slightly lower specificity (80.6% versus 87.5%) (6). Meanwhile, the p16/*K_i_*-67 dual-stained cytology was found to have higher sensitivity than the E6 oncoprotein test (55.2% versus 31.3%) but somewhat lower specificity (80.6% versus 83.6%) (6). p16/*K_i_*-67 dual staining is effective, but it cannot be used to triage self-collected samples as reflex cytology is not possible (31).

The relationship between HPV16 E6 genome variations and the persistent viral infection and an increased risk of developing precancerous lesions and invasive CC had been widely reported and discussed. In France, Grodzki et al. (32) found that the HPV16 E6-350G variant presented an increased risk for development of cervical carcinoma, both for persistence (OR = 3.0; 95% CI: 1.4–6.7) and progression (OR = 6.2; 95% CI: 2.7–14.3). In Mexico, compared with the E-prototype, Ortiz-Ortiz (33) observed that AA-a showed a higher risk of developing CC. Mirabello (34) and Hang (35) found that the appearance of A4 variants among HPV16 positive women conferred a remarkably increased chance of gaining CC. These studies indicated that some HPV16-E6 variants might have a higher oncogenic potential.

Further analysis of the 13 mutation features identified by the Lasso algorithm revealed the significant differences in D32E, H85Y, L90V, and Q98K mutations between HSIL and NHSIL patients in our study. Except for D32E, the mutation frequency of other sites was higher in NHSIL patients (Table S3). Intriguingly, those E6 mutations were all located near the E6-E6AP or E6-p53 interface (Fig. 4), which might affect the E6‐p53 interaction and ultimately cause p53 degradation.

As compared to the prototype, Hadami et al. (36) found that the highest p53 degradation was exhibited by the African variants Af2-a/r, Af1-d/G295, and Af2-a/G285, followed by the European variants E-C442/G350, E-G350/r, and NA1-b/r (*P* < 0.05). Moreover, Cuninghame et al. (37) found that the HIF-1α protein level and activity were increased by the Asian-American E6 (AAE6) variant through augment mitogen-activated protein kinase/extracellular related kinase signaling, leading to a hypoxia-tolerant phenotype with enhanced migratory potential. These studies suggest that HPV16-E6 mutation has biological effects on p53 degradation and metabolic reprogramming, which may play an important role in the occurrence and development of cervical cancer. However, in previous studies on the degradation of p53 by E6 variants, these variants often contain multiple mutation sites, which cannot accurately evaluate the contribution of a single mutation site to the degradation of p53. In this study, we found that the D32E and H85Y variants exhibited a significantly higher ability to degrade p53 compared to the E6 wild type for first time (*P* < 0.05). However, it is noteworthy that, the same as the previous studies (18, 38), the mutation frequency of H85Y is higher in NHSIL patients, indicating that additional mechanisms may be involved in the epithelial transformation process.

Besides D32E and H85Y, we also identified some significant difference mutation sites including L90V, Q98K, and R131K between HSIL and NHSIL; the functions of these different missense mutations need to be further investigated. In addition, for more detailed estimating on the mutation rate of HPV16 and the accumulation of HPV16 variants in patients over time, we still need to conduct a longitudinal study in a larger sample of HPV16-infected populations.

In conclusion, the NGS-based HPV16 E6 sequence analysis in this study revealed more important genetic feature patterns between HSIL and NHSIL patients, highlighting the potential value of this test as a triage test in HPV screening programs. Combining HPV16 mutation patterns with NGS and machine learning methods may help to facilitate HSIL risk assessment and provide highly specific targets in etiology and treatment research. Moreover, to the best of our knowledge, this study is the first to find that the D32E and H85Y variants exhibited a significantly higher ability to degrade p53, which may play a vital role in the development of cervical cancer.

## MATERIALS AND METHODS

### Study population.

From September 2020 to August 2021, a total of 199 DNA samples from HPV16-positive cervical specimens were collected and sequenced by NGS. Based on cytological and histological evaluations of fresh specimens, the cervical lesions were graded according to their severity as follows: 18 nonneoplastic/no evidence of disease (NED; chronic cervicitis and inflammation‐related regenerative changes), 31 low-grade squamous intraepithelial lesion (LSIL) and CIN I, 29 CIN II/III, and 121 CC. The histological diagnosis of each case was reviewed by an experienced pathologist who was unaware of the HPV testing results. Histopathological findings were divided into a high-grade squamous intraepithelial lesion (HSIL, including CIN II/III and CC) group or a non-HSIL (NHSIL, including NED and LSIL) group.

### Study design.

**FIG 6:**
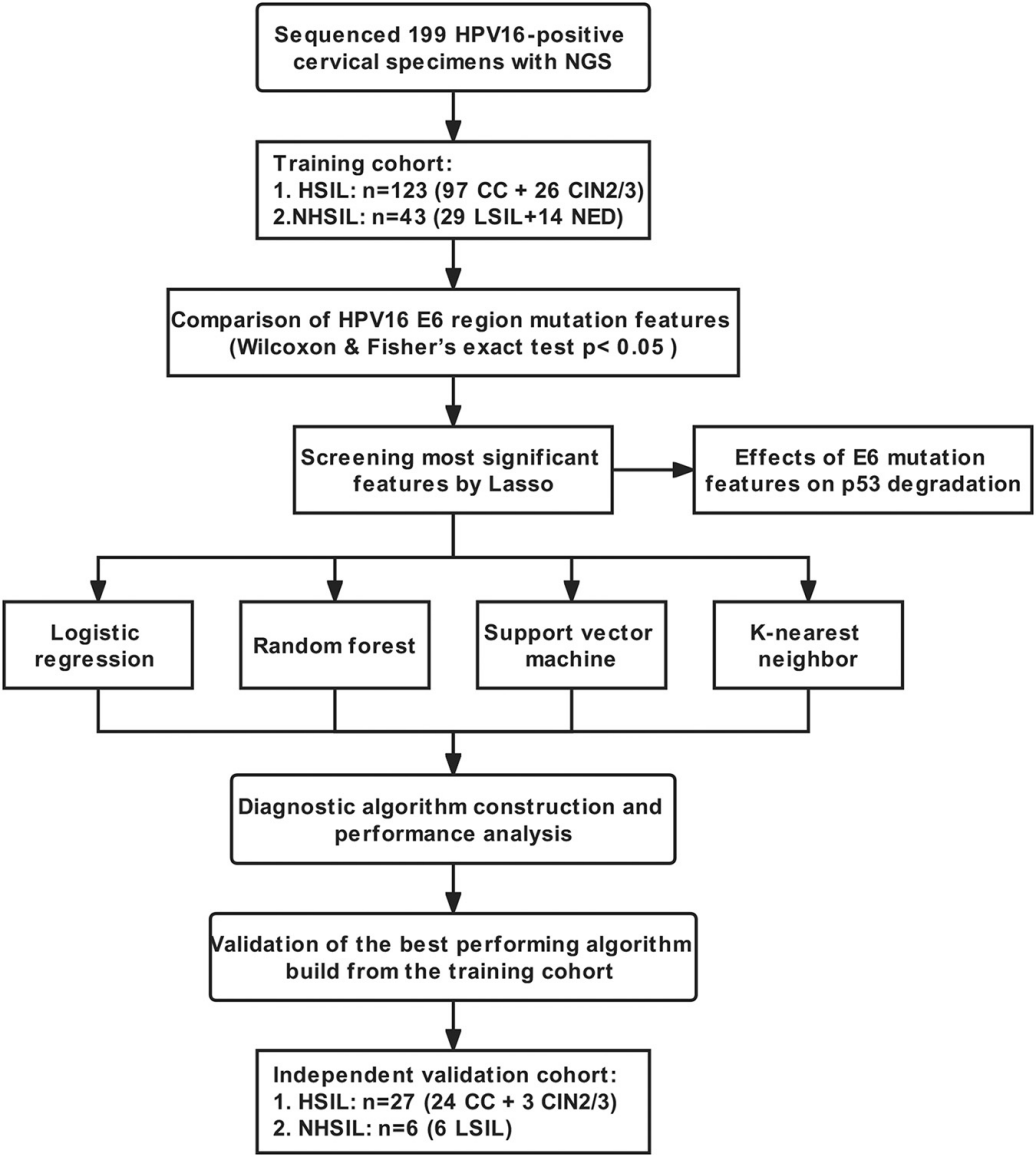
Study design and flowchart. A total of 166 DNA samples from HPV16-positive cervical specimens were collected and divided into HSIL and NHSIL groups for model training. In the independent validation cohort, 33 HPV16-positive cervical specimens were collected for subsequent analysis. CC, cervical cancer; CIN, cervical intraepithelial neoplasia; LSIL, low‐grade squamous intraepithelial lesion; NED, nonneoplastic/no evidence of disease; LR, logistic regression; RF, random forest; SVM, support vector machine; KNN, K-nearest neighbor.

The study was designed to reveal and assess the performance of the HPV16 E6 sequence features based on NGS in the identification and prediction of HSIL outcomes (Fig. 6) as follows.

Training phase: A total of 166 DNA samples from HPV16-positive cervical specimens were collected and divided into HSIL and NHSIL groups for model training. The aa mutation features of HPV16 E6 in HSIL and NHSIL patients were compared by Wilcoxon and Fisher’s exact tests (*P* < 0.05). Then, the Lasso algorithm was used to select the most significant aa mutation features of HPV16 E6. Finally, the logistic regression (LR), random forest (RF), support vector machine (SVM), and K-nearest neighbor (KNN) were used to construct the diagnostic algorithms. The area under the receiver operating characteristics (AUC) curve was used to evaluate their diagnostic performance through 5-fold cross-validation.

Independent validation phase: In the independent validation cohort, 33 HP16-positive cervical specimens were collected to verify the algorithm with the best performance during the training phase.

### Genomic DNA isolation, HPV typing, and sequencing.

The supernatants were removed by centrifugation at 13,000 rpm for 10 min, and the pellets were collected for DNA extraction. Genomic DNA was extracted by nucleic acid extraction reagent (MCP-16C; Yaneng Biotechnology, Shenzhen, China). Human papillomavirus genotyping was conducted using a human papillomavirus genotyping Kit (Yaneng Biotechnology, Shenzhen, China).

After HPV testing, the remaining DNA samples were stored at −80°C. The HPV16 E6 region was amplified using Phusion High–Fidelity DNA polymerase (Thermo Fisher Scientific Baltics UAB, Vilnius, Lithuania) with specific primers. The following primers were used: 5′‐AAACTAAGGGCGTAACCGAAATC‐3′/5′‐CAGCCTCTACATAAAACCATCCAT‐3′, and 5′-CAAGGAGACAGTTTATGCACCA‐3′/5′-TGCAACAAGACATACATCGACC‐3′. Subsequently, for each HPV16-DNA of samples, the HPV16 E6 region (nucleotides 7125–7566) sequencing was performed on the MiSeq sequencer with the MiSeq reagent kit, v3 (Illumina, San Diego, CA, USA) using Illumina paired-end sequencing protocols (Fig. S2). The procedures are described in the supplemental Materials and Methods.

### Cell culture and transfection.

The HPV-negative C-33A cell line was obtained from Cell Bank of the Chinese Academy of Sciences. The cell line was grown in MEM (Gibco by Invitrogen, Carlsbad, CA, USA) containing 10% fetal bovine serum (FBS; Gibco by Invitrogen, Carlsbad, CA, USA) and maintained at 37°C under humidified air and 5% CO^2^. The cell line was authenticated using STR profiling within the last 3 years, and all experiments were performed with mycoplasma-free cells. The pcDNA3.1 vector carrying the HPV16-E6 wildtype (NP_041325.1), and the D32E and H85Y variants, were constructed by HANBIO (Shanghai, China). All plasmid transfections were conducted with Lipofectamine 3000 (Invitrogen, Life Technologies, Carlsbad, CA, USA) according to the manufacturer’s instructions. The transfected cells were further cultured in medium supplemented with 500 μg/mL G418 (Solarbio, Beijing, China).

### Cell proliferation assay.

C-33A cell line transfected with empty pcDNA3.1 and pcDNA3.1 constructs containing the E6 wild type/variants were seeded in 96-well plates at a density of 2 × 10^4^ cells per well. After 24, 48, 72, 96, and 120 h postseeding, 10 μL enhanced cell counting kit 8 (CCK8; Beyotime, Shanghai, China) was added to each well, and the cells were incubated for 2h at 37°C. The absorbance at 450 nm was measured using a microplate reader (Bio-Tek, VT, USA).

### Western blots.

Protein extraction was performed with a RIPA buffer mixture (Beyotime, Shanghai, China), and the proteins were then measured with a BCA protein assay kit (Solarbio, Beijing, China). The quantified samples were run on SDS-PAGE gels and were transferred onto PVDF membranes (Millipore, USA) using a transfer device. The membrane was blocked with 5% skim milk for 2h at room temperature, incubated with the primary antibody at 4°C overnight, and then probed with secondary antibody for 2 h. The target proteins were visualized by the Odyssey imaging system (LI-COR, USA). Antibodies specific for p53 (CST 2524), Flag (CST 8146), and GAPDH (Proteintech 60004-1) used for Western blot analysis were purchased from Cell Signaling Technology (Beverly, MA, USA) and Proteintech (Rosemount, IL, USA).

### Data processing and analysis.

Raw reads from a Miseq sequencer were processed by Cutadapt 3.5 to cut adaptor sequences and trim low-quality reads (base quality Q20). Filtered read pairs were aligned to the HPV16 reference genome sequence (accession number: NC_001526.4) using BWA 0.7.17.

The complexity of each aa position in the E6 region was calculated based on mutation features established by our previous research (23, 24). Mutations were identified and analyzed using R scripts. High mutation frequency was defined as a mutation rate of ≥5% of the total reads in each position. Statistical significance was evaluated using the unpaired Wilcoxon test and Fisher’s exact test to identify differential mutations between the HSIL and NHSIL groups.

The mutation features of HPV16 E6 were shown by the BioCircos.js tool (39). The 3D structure of the HPV16 E6 protein was predicted based on PDB 4xr8 and 4yoz via PyMol 2.5.2 (25, 40). HPV16 E6 features models were trained to discriminate HSIL from NHSIL patients using the four machine learning approaches. The packages of “glmnet,” “caret,” “randomForest,” “e1071,” and “kknn” were conducted to calculate Lasso, LR, RF, SVM, and KNN, respectively, using R version 4.1.2 software. The predictive performance was measured by the receiver operating characteristic (ROC) curves. The ROC curve was used to calculate the optimal cutoff values that were determined by maximizing the Youden index. Accuracy of the optimal cutoff value was assessed by sensitivity, specificity, positive predictive value (PPV), and negative predictive value (NPV).

### Ethics approval.

The study was approved by the Institutional Ethics Committee of the leading medical center (Shanghai Eastern Hepatobiliary Surgery Hospital, EHBHKY2020-02-012). Written informed consent was obtained from all participants.

### Data availability.

The sequencing data used in this study have been submitted to NCBI under BioProject PRJNA830986.
