# Supplementary material for: Deep Sequencing of HPV16 E6 Region Reveals Unique Mutation Pattern of HPV16 and Predicts Cervical Cancer
Source: Microbiol Spectr. 2022 Jun 23;10(4):e01401-22. doi: 10.1128/spectrum.01401-22 (PMC9430801; doi:10.1128/spectrum.01401-22)
Supplement: Supplemental file 1 — Supplemental material. Download spectrum.01401-22-s0001.pdf, PDF file, 0.4 MB [file spectrum.01401-22-s0001.pdf]

Supplementary Materials and Methods

Contents

Illumina paired-end sequencing protocols of HPV16 E6 region .... - 1 -

1、HPV DNA extraction .... - 1 -

2、PCR amplification of HPV16 E6 fragment .... - 1 -

3、DNA agarose gel electrophoresis .... - 3 -

4、Extraction of DNA fragments from gels .... - 3 -

5、DNA fragment sorting and purification .... - 4 -

6、DNA quantification and mixing .... - 5 -

7、Next-generation sequencing library construction .... - 5 -

8、Library qPCR .... - 6 -

9、Next-generation sequencing of HPV16 E6 fragment .... - 7 -

10、Sequencing data analysis process .... - 7 -

Supporting Information Table .... - 9 -

Supporting Information Figures .... - 12 -

Illumina paired-end sequencing protocols of HPV16 E6 region

1、HPV DNA extraction

(1) Take out MCP-16C reagent plate and turn it up and down several times to make magnetic beads resuspended and mixed. Then, centrifugation at 500 rpm for 1minute.

(2) Take 300 μl of mixed sample and add it to the first or seventh columns of MCP-16C reagent plate. Then put the reagent plate and magnetic bar into the corresponding card slot and automate extraction according to the following settings:

Step	Position (columns)	Function	Time (min)	Mixing time (min)	Magnetic time (min)	Temp (℃)
1	2、8	Magnetic bead transfer	0	0	1	25
2	1、7	Lysis	0	15	1	25
3	3、9	Washing I	0	1	1	25
4	4、10	Washing II	0	1	1	25
5	5、11	Elution	5	5	1	25
6	2、8	Magnetic bead	0	1	0	25

(3) After the procedure, the extracted DNA was located in column 5 and column 11.

2、PCR amplification of HPV16 E6 fragment

(1) DNA extracted from MCP-16C was used as template for PCR amplification with E6 specific primers.

Primer information:

Name		Sequence (5'-3')
NEST	Forward	AAACTAAGGGCGTAACCGAAATC
	Reverse	CAGCCTCTACATAAAACCATCCAT
E6	Forward	CAAGGAGACAGTTTATGCACCA
	Reverse	TGCAACAAGACATACATCGACC

PCR reaction component:

Component	Volume (μl)	Final concentration

H_2O	28.5	
5X Phusion™ HF Buffer	10	1X
2.5 mM dNTPs	4	200 μM each
Forward primer	2.5	0.5 μM
Reverse primer	2.5	0.5 μM
Template DNA	2	
Phusion™ High–Fidelity DNA Polymerase	0.5	0.02 U/μl
Total volume	50	

PCR reaction condition:

Temperature	Time	Cycles
95℃	1 min	
98℃	30 s	
98℃	10 s	
63℃	1 min	35 cycles
72℃	1 min	
72℃	7 mins	
4℃	Hold	

(2) Different samples were combined with specific primers of different Barcode combinations, and the results of the first round of amplification were used as the template for the second round of PCR amplification of E6 region.

PCR reaction component:

Component	Volume (μl)	Final concentration
H_2O	29.5	
5X Phusion™ HF Buffer	10	1X
2.5 mM dNTPs	4	200 μM each
Forward primer	2.5	0.5 μM
Reverse primer	2.5	0.5 μM
Template DNA	1	

Phusion™ High–Fidelity DNA Polymerase	0.5	0.02 U/μl
Total volume	50	

PCR reaction condition:

Temperature	Time	Cycles
95℃	1 min	
98℃	30 s	
98℃	10 s	
63℃	1 min	35 cycles
72℃	30 s	
72℃	7 mins	
4℃	Hold	

3、DNA agarose gel electrophoresis

(1) Mix 1.5 g agarose and 100 ml 1×TAE buffer solution in a conical flask, seal with filter paper and heat in microwave oven.

(2) When the agarose is completely melted, the flask is cooled at room temperature to about 50℃.

(3) Add Ethidium bromide (EB) to the flask, mix well, and immediately pour it into a gel box with a comb.

(4) After the gel has solidified, remove the comb and gel and place the gel in a horizontal electrophoresis tank containing 1×TAE buffer.

(5) Add 6×Loading Buffer to the PCR amplification product so that the final concentration is 1×

(6) After mixing, the samples were carefully added to the loading wells of the gel, and the samples were observed and photographed with UV after electrophoresis at 140V for 27 minutes.

4、Extraction of DNA fragments from gels

(1) Excise the DNA fragment from the agarose gel with a clean, sharp scalpel. Minimize the size of the gel slice by removing extra agarose.

(2) Weigh the gel slice in a colorless tube. Add 3 volumes of Buffer QG to 1 volume of gel (100 mg, or approximately 100 μl).

(3) Incubate at 50°C for 10 minutes or until the gel slice has completely dissolved. To help dissolve gel, mix by vortexing the tube every 2-3 minutes during the incubation.

(4) After the gel slice has dissolved completely, check that the color of the mixture is yellow (similar to Buffer QG without dissolved agarose). If the color of the mixture is orange or violet, add 10 μl of 3 M sodium acetate, pH 5.0, and mix. The color of the mixture will turn to yellow.

(5) Add 1 gel volume of isopropanol to the sample and mix.

(6) Place a QIAquick spin column in a provided 2 ml collection tube.

(7) To bind DNA, apply the sample to the QIAquick column, and then centrifuge for 1 minute.

(8) Discard flow-through and place QIAquick column back into the same collection tube.

(9) To wash, add 0.75 ml of Buffer PE into the QIAquick column and centrifuge for 1 minute.

(10) Discard the flow-through and centrifuge the QIAquick column for an additional 1 minute at 7,900g (13,000 rpm).

(11) Place QIAquick column into a clean 1.5 ml microcentrifuge tube.

(12) To elute DNA, add 50 μl of Buffer EB (10 mM Tris·Cl, pH 8.5) to the center of the QIAquick membrane, and then centrifuge the column for 1 minute.

5、DNA fragment sorting and purification

	HPV16-E6
Volume ratio of the first round (Beads : DNA)	0.55×
Volume ratio of the first round (Beads : DNA)	0.15×

Note: "×" in the table indicates the sample DNA volume.

(1) Take the Hieff NGS® DNA Selection Beads out of the refrigerator, equilibrate at room temperature for 30 minutes, and then vortex to mix the beads.

(2) Add the first round of magnetic beads to the DNA solution for sorting according to the table above, mix the magnetic beads and incubate at room temperature for 5 minutes.

(3) The EP tube was microcentrifuged and placed in a magnetic stand. After the solution was clear, the supernatant was aspirated and placed in another EP tube.

(4) According to the above table, add the second round of magnetic beads to the supernatant for sorting, mix the magnetic beads evenly, and stand at room temperature for 5 minutes.

(5) The EP tube was microcentrifuged and placed on a magnetic stand for about 5 minutes. After the solution became clear, the supernatant was removed.
(6) Rinse the magnetic beads with 200 μl of fresh 80% ethanol and then incubate at room temperature for 30 seconds to discard the supernatant (the centrifuge tube is located in the magnetic rack)。
(7) Repeat step (6)。
(8) Open the EP tube cover for about 5 minutes, and wait for the magnetic beads to dry until cracks appear (do not dry for too long).
(9) Take out the EP tube from the magnetic stand and add an appropriate amount of ddH2O (≥20 μl), and mix well by pipetting up and down, and then incubate at room temperature for 5 minutes.
(10) The EP tube was microcentrifuged and placed in a magnetic stand for about 5 minutes. After the solution became clear, the supernatant was transferred to another clean EP tube to complete the sorting.

6、DNA quantification and mixing

(1) Prepare an appropriate amount of 0.5 ml PCR thin-walled tube and mark it at the tube cap.
(2) Dilute dsDNA Reagent to 1× with dsDNA Buffer (use within 3 hours).
(3) Add 190 μl of quantitative detection working solution to the standard PCR tube, then add 10 μl of dsDNA standard1 and dsDNA standard2 to the corresponding standard PCR tube, and mix by vortexing.
(4) Add 199 μl of quantitative detection working solution to the sample PCR tube, add 1 μl of the sample and vortex for 2-3 seconds.
(5) After incubating for 2 minutes at room temperature in the dark, place the PCR tube to be tested into the Qubit fluorometer and select a high-sensitivity dsDNA detection program to detect the fluorescence signal value.
(6) Combine sorted and purified products in equal moles.

7、Library construction

(1) Take 50 ng of mixed DNA, and use different Index to construct PCR library.

PCR reaction component:

Component	Volume (μl)	Final concentration
H2O	Add to 50 μl	

5X Phusion™ HF Buffer	10	1X
2.5 mM dNTPs	4	
Illumina F	1	10 μM
Index	1	10 μM
Template DNA	50 ng	
Phusion™ High–Fidelity DNA Polymerase	0.5	
Total volume	50	

PCR reaction condition:

Temperature	Time	Cycles
98°C	30 s	
98°C	10 s	
60°C	30 s	10 cycles
72°C	1 min	
72°C	7 mins	
4°C	Hold	Hold

(2) The amplified library was sorted and purified using Hieff NGS® DNA Selection Beads, as in step 5.

(3) Use Qubit to quantify the DNA of the sorted and purified library, the method is the same as step 6.

8、Library qPCR

(1) Take the library after sorting and purification in step 7 and dilute it by gradient (200 pM, 20 pM and 4 pM), and then perform qPCR quality inspection on the library.

qPCR reaction component:

Component	Volume (μl)	Final concentration
H2O	16	
SYBY Green Realtime PCR Master Mix	25	
F	2	10 μM
R	2	10 μM

Template DNA	5
Total volume	50

qPCR reaction condition:

Temperature	Time	Cycles
95°C	1min	
95°C	15s	
60°C	15s	40 cycles
72°C	45s (Data collection)	
Melt Curve Analysis		
4°C	Hold	

9、Next-generation sequencing of HPV16 E6 fragment

HPV16 E6 region (nt7125-7566) sequencing was performed on the MiSeq sequencer with the MiSeq Reagent Kit, v3 (Illumina, San Diego, CA, USA).

(1) Add 5 µl of freshly prepared 0.2 N NaOH and 5 µl of the diluted and mixed library to a 1.5 ml centrifuge tube, and centrifuge at 280g at room temperature for 1min after mixing.

(2) The centrifuged samples were allowed to stand at room temperature for 5 minutes to fully denature DNA.

(3) Add 990 µl HT1 to the library in the previous step and invert the sample 10 times to mix the samples. Set aside at 4°C.

(4) Scrub the sequencer chip well with isopropanol and let it dry.

(5) Use lens tissue to wipe the flow cell rinsed with deionized water, then load it into the chip slot of the sequencer, and close the compartment door after the flow cell is successfully read in.

(6) Inject the mixed library into the Load Samples slot, load the PR2 bottle, and empty the waste bottle.

(7) Load the kit and start sequencing.

10、Sequencing data analysis process

The original sequencing data was processed by removing adapters, quality control, alignment and ligation to obtain a clean sample for subsequent analysis.

(1) The quality of the raw sequencing data was assessed using FastQC0.11.9, and information such as the

quality of each base, sequencing depth, GC content and adapter content was analyzed. **(Figure S1)**

(2) Use Cutadapt3.5 to remove adapter sequences from each read and remove reads with sequencing end quality less than Q20.

(3) The filtered reads were aligned to the HPV16 reference sequence (NC_001526.4) using bwa0.7.17.

(4) Calculation of mutation characteristics: Using the NCBI HPV16 reference sequence as a control, R4.1.2 was used to analyze the amino acid mutations in the E6 region of HPV16.

Supporting Information Table

TABLE S1 Baseline characteristics of HSIL and NHSIL patients.

Cohort			Age (years)	
			Median	Range
**Training cohort (n=166)**	HSIL (n=123)	CC (n=97)	53	26-81
		CIN II/III (n=26)	37	24-59
	NHSIL (n=43)	CIN I/LSIL (n=29)	37	26-55
		NED (n=14)	33	21-45
**Independent validation cohort (n=33)**	HSIL (n=27)	CC (n=24)	52	31-69
		CIN II/III (n=3)	33	32-56
	NHSIL (n=6)	CIN I/LSIL (n=2)	40	39-41
		NED (n=4)	38	35-49

Note: CC, cervical carcinoma; CIN, cervical intraepithelial neoplasia; LSIL, Low grade squamous intraepithelial lesion; HSIL, High grade squamous intraepithelial lesion; NED, no evidence of disease

TABLE S2 Twelve E6 mutation sites have a higher average mutation frequency in HSIL patients.

Region	Position	WT	Average Mutation Frequency		UP/DOWN
			HSIL	NHSIL	
E6	1	M	0.00363	0.003391	UP
E6	5	R	0.015543	0.011136	UP
E6	8	M	0.003406	0.003214	UP
E6	12	P	0.007439	0.00635	UP
E6	17	R	0.044813	0.013935	UP
E6	27	Q	0.009579	0.005787	UP
E6	32	D	0.84681	0.480558	UP
E6	36	E	0.015715	0.011381	UP
E6	37	C	0.012003	0.005676	UP
E6	100	N	0.014713	0.00984	UP
E6	133	H	0.014655	0.009128	UP
E6	144	M	0.004323	0.003002	UP

Note: HSIL, High grade squamous intraepithelial lesion; NHSIL, Non-high grade squamous intraepithelial lesion; WT, Wildtype (HPV16 reference genome sequence NC_001526.4)

TABLE S3 The difference E6 missense mutations between the HSIL and NHSIL patients.

Variant	Average Mutation Frequency		P value	
	HSIL	LSIL	Wilcoxon test	Fisher's exact test
D32E	0.833852	0.458324	6.11E-06	1.34E-05
H85Y	0.044148	0.295897	0.000178	1.70E-07
L90V	0.032479	0.107557	1.56E-07	0.008929
Q98K	0.007078	0.029113	8.33E-11	0.000591
R131K	0.005298	0.018514	0.00459	0.039604

TABLE S4 The difference E6 mutations among subgroups of the HSIL and NHSIL patients.

Variant	HSIL (CC vs CIN2/3)		NHSIL (LSIL vs NED)	
	p_value(wilcox)	p_value(fisher)	p_value(wilcox)	p_value(fisher)
R62I	1.99E-06	0.000304	0.184247	1
L90V	1.46E-06	0.000159	0.389782	0.291435
Q98K	1.15E-11	0.000304	0.029215	0.703691
E120D	0.00165	0.030289	0.023666	0.038714
Q130R	9.15E-08	0.008592	5.28E-06	0.286103
R131K	0.005465	0.043316	1.94E-05	0.286103

Note: CC, cervical carcinoma; CIN, cervical intraepithelial neoplasia; LSIL, Low grade squamous intraepithelial lesion; HSIL, High grade squamous intraepithelial lesion; NED, no evidence of disease

Supporting Information Figures

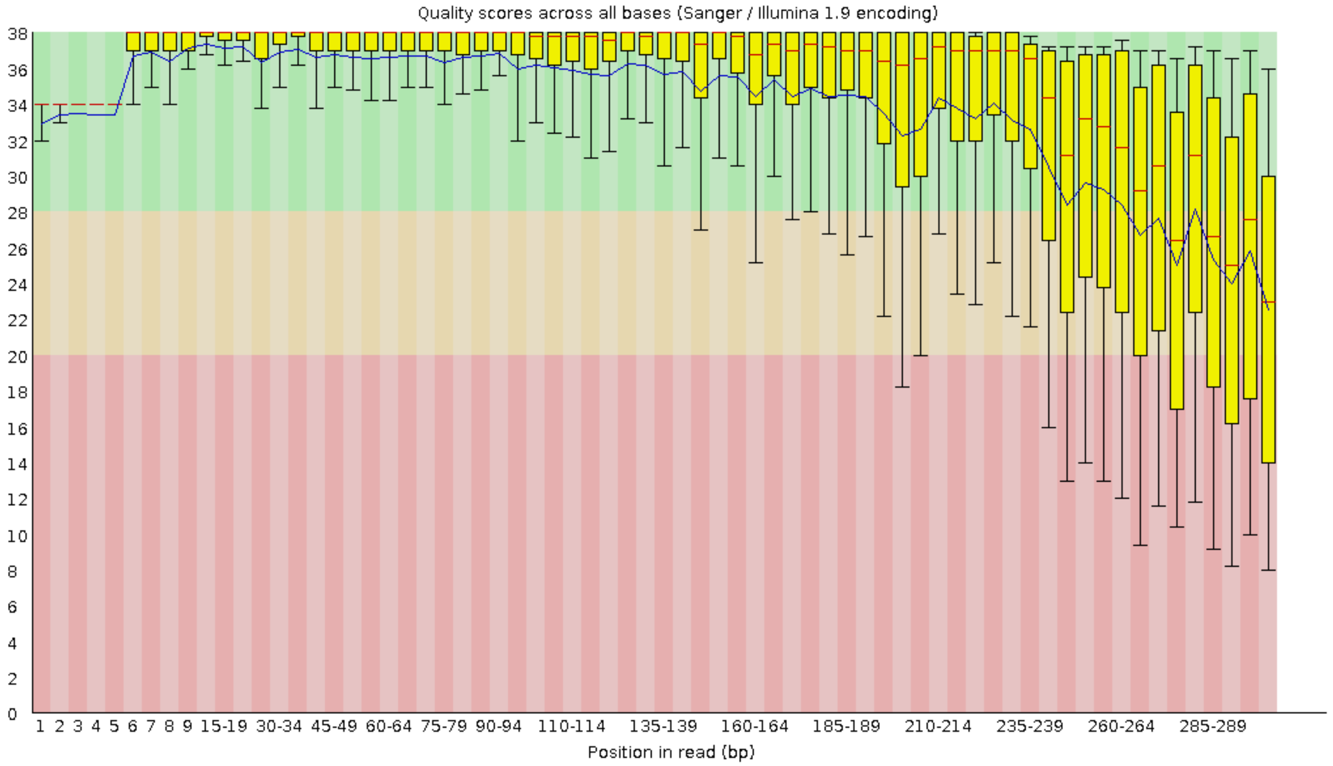

FIG S1 Quality scores all bases (Sanger/Illumina 1.9 coding) of HBV RT region.

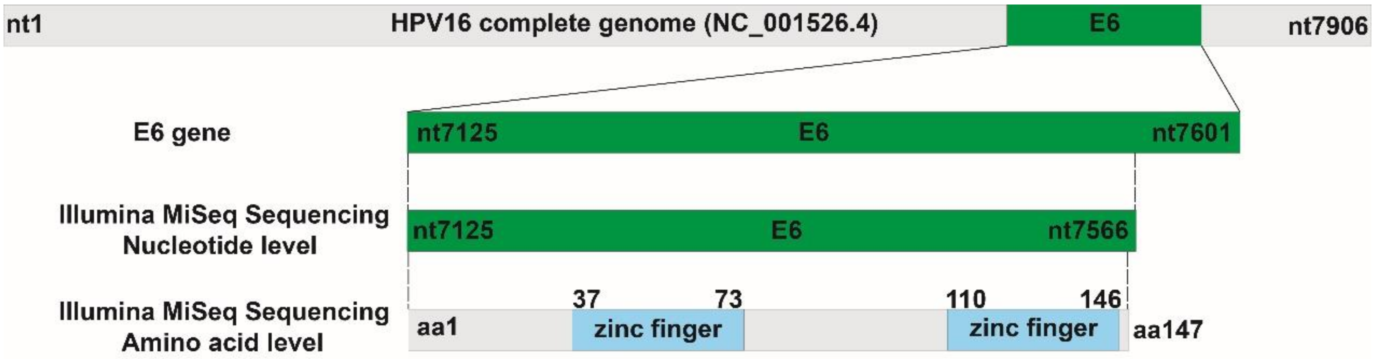

FIG S2 Schematic diagram of HPV16 E6 sequencing.

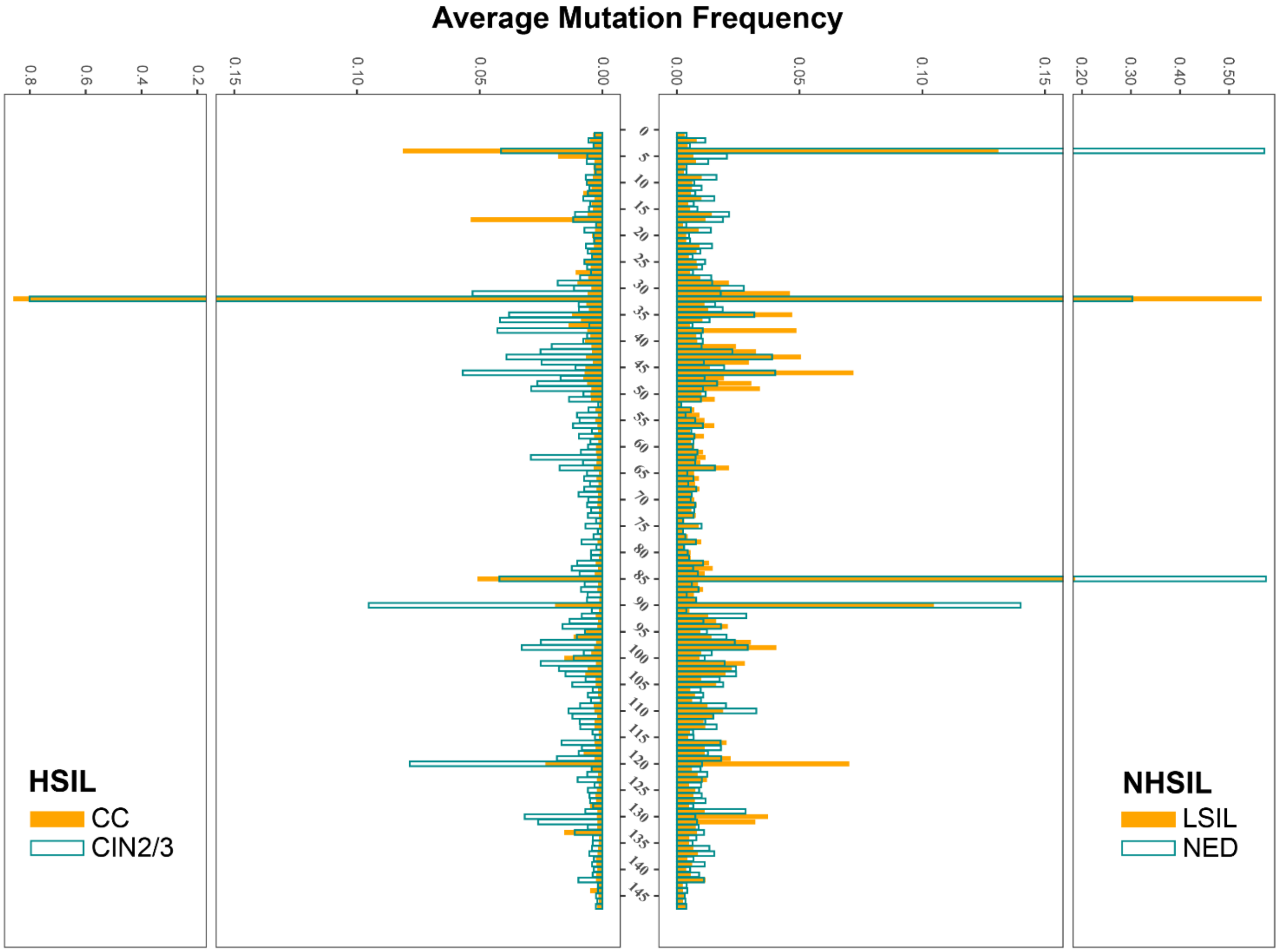

FIG S3 The complexity of HPV16 E6 region between the HSIL and NHSIL subgroups.

The barplot demonstrates differential average mutation frequency of each position in the HPV16 E6 aa1-147 region between the HSIL and NHSIL subgroups. Note: CC, cervical carcinoma; CIN, cervical intraepithelial neoplasia; LSIL, Low grade squamous intraepithelial lesion; HSIL, High grade squamous intraepithelial lesion; NED, no evidence of disease
